# A biomimicry design for nanoscale radiative cooling applications inspired by *Morpho didius* butterfly

**DOI:** 10.1038/s41598-018-35082-3

**Published:** 2018-11-15

**Authors:** Azadeh Didari, M. Pinar Mengüç

**Affiliations:** Center for Energy, Environment and Economy (CEEE), Özyegin University, Istanbul, 34794 Turkey

## Abstract

In nature, novel colors and patterns have evolved in various species for survival, recognizability or mating purposes. Investigations of the morphology of various butterfly wings have shown that in addition to the pigmentation, micro and nanostructures within the wings have also allowed better communication systems and the pheromone-producing organs which are the main regulators of the temperature within butterfly wings. Within the blue spectrum (450–495 nm), *Morpho didius* butterfly exhibit iridescence in their structure-based wings’ color. Inspired by the rich physics behind this concept, we present a designer metamaterial system that has the potential to be used for near-field radiative cooling applications. This biomimicry design involves SiC palm tree-like structures placed in close proximity of a thin film in a vacuum environment separated by nanoscale gaps. The near-field energy exchange is enhanced significantly by decreasing the dimensions of the tree and rotating the free-standing structure by 90 degrees clockwise and bringing it to the close proximity of a second thin film. This exchange is calculated by using newly developed near-field radiative transfer finite difference time domain (*NF-RT-FDTD*) algorithm. Several orders of enhancement of near-field heat flux within the infrared atmospheric window (8–13 μm bandwidth) are achieved. This spectrally selective enhancement is associated with the geometric variations, the spatial location of the source of excitation and the material characteristics, and can be tuned to tailor strong radiative cooling mechanisms.

## Introduction

Spectrally tunable thermal emitters are essential to achieve radiative cooling. Such emitters are designed to radiate strongly within the infrared atmospheric transparency window (8–13 μm) and to reflect strongly within the solar spectrum. Development of these emitters might be possible by bringing together the right combination of materials, unique photonic and geometric features. Although nature provides abundant amount of materials and structures, controlling their functionality with the ‘designer’ metamaterials is crucial for the development of robust and reliable new technologies.

In recent years, different materials have been proposed and used in order to achieve radiative cooling; they include, composite^1,2^ and polymeric^3,4^ materials, white paints^5,6^, and Silicon monoxide films^7–9^, which are shown to increase radiative emission within the atmospheric window. However, almost all of these materials either lack high emissivity or exhibit narrowband emission within this window^1,2,5–10^. Moreover, many of these materials undesirably have high absorption within the solar spectrum, rather than high reflectance, which does not allow the structures to cool down below the environment temperature^1,3–5,11^. Alternatively, it is possible to attain a highly selective broad band absorption with metamaterials^12–16^. Metamaterials are manufactured in the laboratory settings; they are composed of periodic, macroscopic structures that could be tuned to achieve specific electromagnetic properties^17^. Several researchers have shown that these man-made materials, including metallic plasmonic nanostructures^18–24^ and photonic crystals^25–28^ display highly selective optical absorption at infrared spectrum. Yet, it is usually difficult to achieve a broadband absorption spectrum. Also, metallodielectric metamaterials have interesting optical characteristics as recently demonstrated by several researchers^14,29–31^.

Nature-inspired solutions have been utilized by many research fields including health, engineering and sustainable environments. Biomimicry designs and biomimetic materials have been employed to creatively tackle some of the most challenging problems of our time. Different insects and species such as butterflies and beetles are considered as the design models used as in micro and nano-photonics for such applications^32^. Biomimicry studies may allow insight to structural variations and features that could be used to design thermal emitters for radiative cooling applications. It was shown, for example, that a biomimetic design inspired by the thermo-regulatory effect of hairs of Saharan silver ant remarkably increases both the optical reflection and mid-infrared emission^33^ which shows that in these organisms a number of factors lead to their unique properties and characteristics.

The research on various species of butterflies has led many groups to have extensive and inspirational biomimicry studies^32^. Innovative ideas can emerge from such studies only if we understand why nature has preferred a particular nano- or micro-scale structure over other alternatives. For example, some butterfly structures have evolved for the development of a unique system of thermal regulators within the wings of butterflies^34^. The evolution has also aimed towards better communication systems and the improvements of pheromone-producing organs. For these organisms to function properly, control of their temperature is essential. Investigations of the morphology of wings have shown that micro- and nano-structures along with the pigment-based colors of butterfly are the main regulators of inhomogeneous distribution of temperature within their wings^34^.

**Figure 1:**
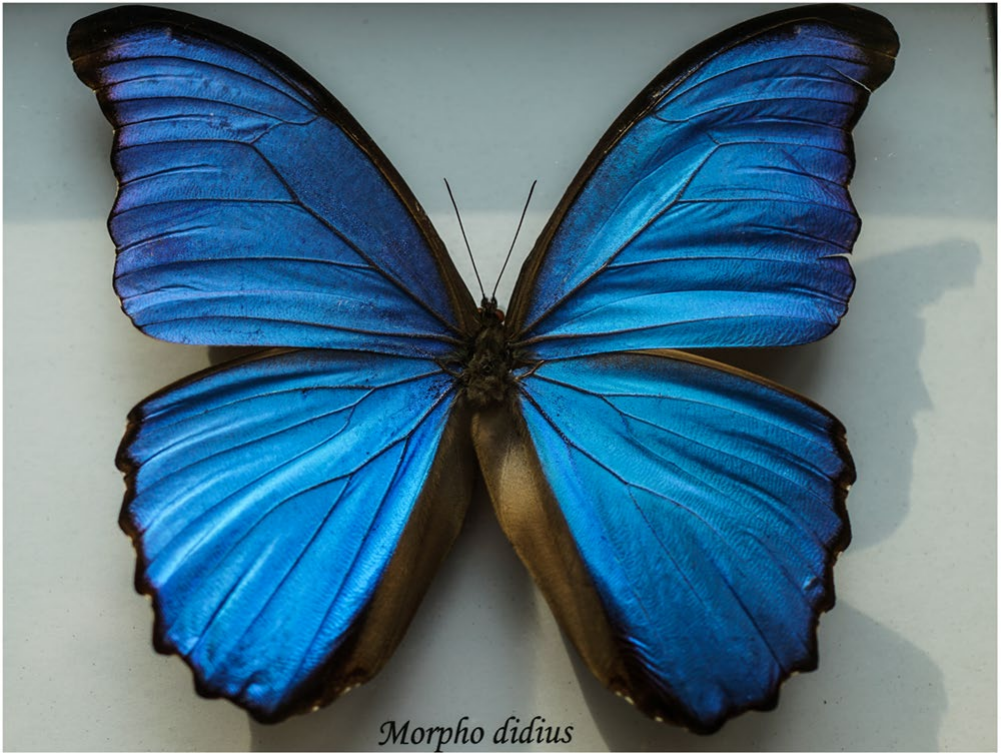
*Morpho didus* butterfly of Peru.

**Figure 2:**
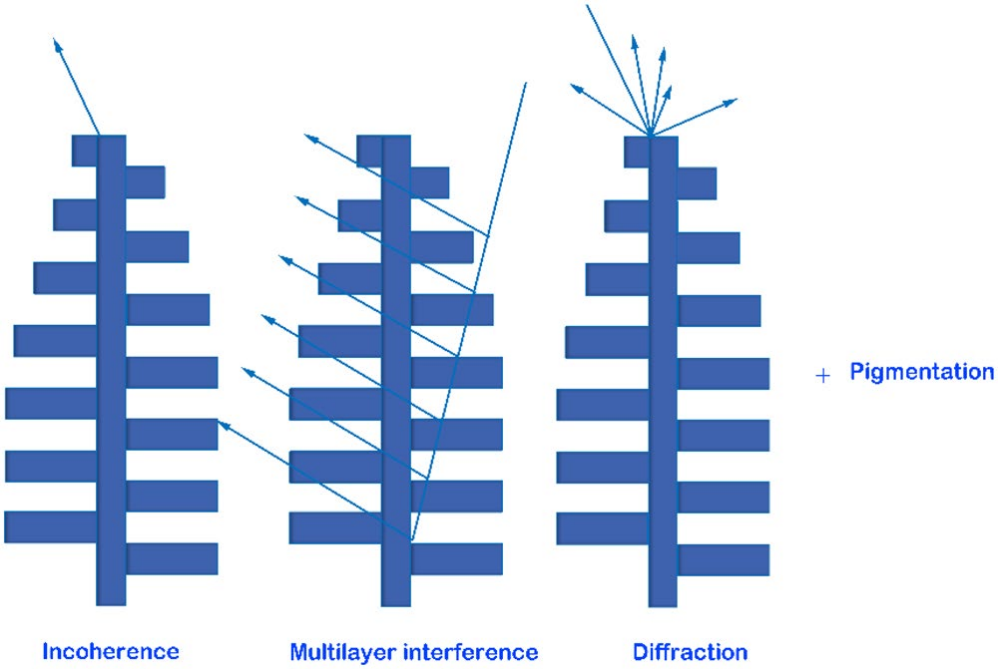
A schematic describing physical light-matter interactions which lead to blue coloring of *Morpho* butterfly wings when a plane wave is incident on different layers of tree-like structures.

Figure 1 shows the intricate colors of a Peruvian *Morpho* butterfly. This iridescent blue color is mainly due to structural effects rather than pigmentation, as discussed by different researchers^35,36^. Iridescence is in essence structural, and the corresponding color is a result of constructive or destructive interference of light propagating through periodic structures. In various species of *Morpho* butterfly, different shades of blue color are observed, which corresponds to 470–500 nm wavelength in the visible spectrum. This narrow-band phenomenon is the results of a number of optical phenomena, which can be summarized as: i) high reflectivity in the blue spectrum due to interference; ii) diffuse reflection is due to the width of the scales and irregular height of the ridges, iii) pigmentation impacts the blue contrast and changes the hue; and iv) the glossy look of the wings which is a result of scales that cover the wings. In short, a combination of structural regularity and irregularity and the interactions of the structural color with pigmentation results in the unique appearance of the *Morpho* colors^35,36^. A schematic showing different physical light-matter interactions which lead to such intricate colors is depicted in Fig. 2.

Inspired with the blue or light-purple iridescence of *Morpho* butterflies, researchers from different backgrounds have developed different fabrication techniques^37–41^. Among many applications, there are those to enhance the efficiency of solar cells^41–43^ and those with the focus on tuning the functionality of the systems within the visible spectrum^44^ are noteworthy. In the present study, we focus on structures which show the potential to be used in mid-infrared wavelength spectrum for radiative cooling applications, which have not yet been thoroughly investigated. Our studies suggest that the development of a metamaterial-based biomimetic emitter with highly reflective structures within the blue spectrum (450–495 nm) is possible, and they have the potential for attaining radiative cooling within the atmospheric window (8–13 μm). In addition, significant enhancement of near-field radiative heat transfer in complex structures can be achieved, as discussed later in the paper.

## Methodology

**Figure 3:**
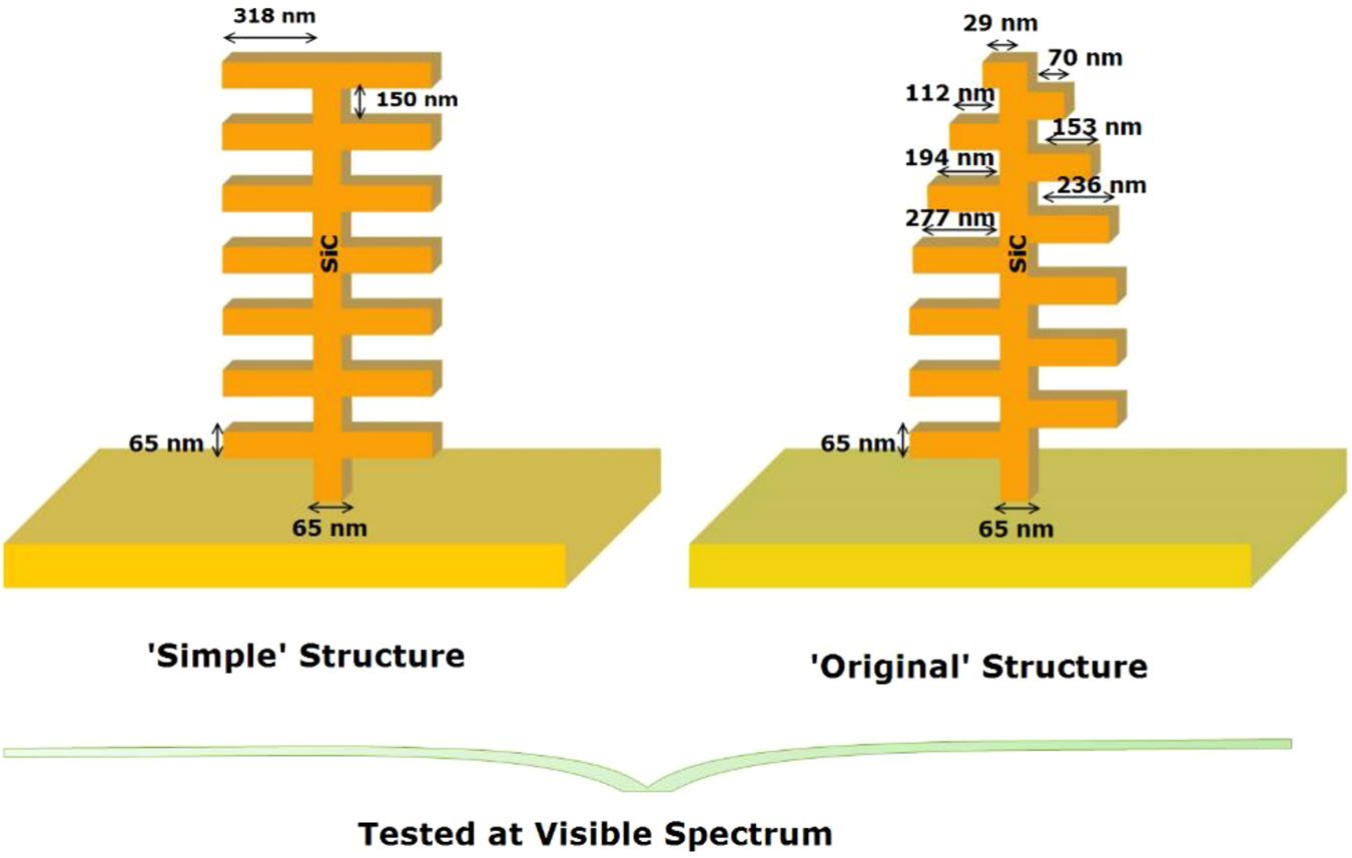
Schematics of ‘simple’ and ‘original’ structures studied at the visible spectrum. Note that the yellow-colored substrate is not considered in the calculations and is solely displayed for the sake of visualization.

The original inspiration for the present work came from Siddique *et al*.^45^ who investigated the nanostructures of blue *Morpho* butterfly. They showed how the reflection spectrum can be controlled by the geometric design of the nanostructures, which is indeed the source of the strong blue iridescence of wings. TEM images of the cross-section of the ground scale of wings revealed what they call “Christmas-tree” (“pine-tree”) like structure is responsible for the well-known blue iridescence. Siddique *et al*.^45^ also explored the source of broad reflection angle of these nanostructures and showed that the structural patterns of alternating branches are among the most important design factors. Particularly, the variations within the structure and the height differences between neighboring branches. Two of the considered design patterns by them are depicted in Fig. 3 and referred to as ‘simple’ structure and ‘original’ structure. They performed a 2D Finite Element Method (FEM) to calculate the reflectance profiles of these structures. The results showed a broadening of the reflection spectrum for the ‘original’ structure, although the intensity peak was the highest for the ‘simple’ structure. The structures the researchers fabricated by e-beam lithography were not free standing; instead they lay flat on a Silicon wafer^45^. Yet, they sustained the intense blue iridescence over a wide angular range.

The main question we ask here is ‘Can we create metamaterials to enhance radiative cooling by using the ideas behind the scales of such butterflies?’ Radiative heat transfer can be categorized into two different categories; The near-field and far-field regimes. In the near-field regime, closely spaced media (where the separation distance between them is much smaller or comparable to the wavelength of light) that support surface waves can give rise to additional enhancements over that for far-field radiative transfer by orders of magnitude. These additional enhancements cannot be predicated when only a single medium is present or if the media are separated by gaps that are greater in length than the wavelength of interest; that is when the radiative heat transfer between them is bounded by the Planck blackbody predictions. Hence, the additional enhancements can only be observed at the near-field regime^46–48^.

Biomimetic designs inspired by various organisms in nature include micro- and nano-structured geometries that can reflect, diffract and scatter light beyond the limits of today’s engineering. They give rise to structural colors in which the exact order and combinations of the scales results in the exhibition of certain colors. The corresponding characteristics show that a simple change in these length scales and ordered structures may cause total destruction of some colors or create a totally different set of colors. The study of structural color has been the focus of many optics-related applications such as wearable technologies. Investigation of these structures, requires utmost accuracy and attention to the details, particularly at nano-scale dimensions, in order to be able to account for all the edge effects and irregularities. For this, effective medium theory-based solutions such as RCWA or MESH^49^ cannot be used for accurate predictions. We need to consider methodologies that not only do not oversimplify these structures, but also are capable of taking into account all the fine nano-scale details.

For the present study, we have chosen the ‘simple’ and ‘original’ structures as the figures of merit. We performed 2D TM mode analysis by using *NF-RT-FDTD* algorithm^50–52^ which is a computational tool specifically designed to evaluate radiative heat transfer at nano-scales. It is capable of taking into account fine details of the arbitrary structures with roughness and asperities accurately. In order to obtain the spatial and spectral characteristics of near-field thermal radiation, we first calculate the local density of states (near-field radiative emission) and then determine the radiative heat flux profiles. Thermal radiation is assumed to be the result of existence of fluctuating electromagnetic fields which themselves are the results of fluctuating electric and magnetic sources within the medium of interest. The fluctuation dissipation theorem (FDT) provides correlation function between different radiation sources.

A combination of FDT and stochastic Maxwell’s equations gives us the required information to study the near-field thermal radiation at nano-scales^53^. These calculations can be carried out using the photonic Green function, *G(r, r′; ω*)^54^. The Green function describes the propagation of energy carriers of angular frequency, *ω*, from the source at point location *r′*, to the observation point at location *r*. It takes into account all the scattering parameters and evaluates the possibility that a photon will arrive at a specific location from a given excitation source. Green function is used in the calculations of local density of electromagnetic states (LDOS) which is necessary to obtain radiative heat flux in near field thermal radiation related problems. The derivations of the required formulations and mathematical considerations can be found in details in our earlier study^52^. Hence, we have avoided repetition of these expressions here.

Drude-Lorentz permittivity expression (provided in Eq. (1)) is used to model the dielectric function of phononic materials such as SiC which was used in this work.

1 $${\varepsilon }_{r}(\omega )={\varepsilon }_{\infty }\frac{{\omega }^{2}-{\omega }_{LO}^{2}+i{\rm{\Gamma }}\omega }{{\omega }^{2}-{\omega }_{TO}^{2}+i{\rm{\Gamma }}\omega }$$

where for silicon carbide *ε*_0 _= 6.7, ${\omega }_{LO}=1.825\times {10}^{14}$ rad/s, ${\omega }_{TO}=1.494\times {10}^{14}$ rad/s, and ${\rm{\Gamma }}=8.966\times {10}^{11}$s^-1^ as given in^55^. Since SiC displays a negative real ${\varepsilon }_{r}(\omega )$ between its transverse ${\omega }_{TO}$ and longitudinal ${\omega }_{LO}$ optical phonon resonance frequencies. The structure is assumed to be excited with a Ricker wavelet right in the middle of the tree trunk and the local density of states and radiative heat flux are recorded at various distances above the tree but we only report here the results observed at 20 nm above the tree. The expression for the Ricker wavelet is given as

2 $${f}_{r}(t)=(1-2{\{\pi {f}_{p}[t-{d}_{r}]\}}^{2})\,exp(-{\{\pi {f}_{p}[t-{d}_{r}]\}}^{2})$$

where *f*_*p*_ is the peak frequency (highest energy), *d*_*r*_ is the temporal delay, defined as *d*_*r*_ = *M*_*d*_/*f*_*p*_, where *M*_*d*_ is the delay multiplier^56^.

The specific geometries of the tree-like structures are shown in Fig. 3. Here we emphasize that we model only the tree structure, which shows the near field effects due to the proximity of branches to each other; the yellow-colored zone is not considered in the calculations and is solely displayed for the sake of visualization.

The ‘original’ structure is assumed to have various dimensions of the branches whereas the ‘simple’ structure is only made of stack of thin films of 65 nm thickness with interbranch separation space of 150 nm. The overall height of a structural-tree is taken as 1505 nm. The Convolutional Perfectly Matched layer (CPML) boundary conditions^56^ were applied at the top and bottom and right and left walls surrounding the structure in order to truncate the physical geometry of the interest and the width of each CPML layer was set to 500 nm. The grid size in both *X* and *Z* directions were set to 5 nm and verification tests were performed to ensure the convergence of the results^52^. The temperature of the emitting structure (tree) was considered as *T* = 1000 K and *T* = 0 K was assumed for the non-emitting thin film. The simulations were performed on a Hewlett-Packard HP-Z820 workstation, equipped with 2 CPUs of 10 physical and 20 logical cores each, with system memory of 32 Gb. This HP-Z820 was also fitted with a workstation-grade Nvidia GPU, the Quadro K4000 that has 3 Gb of very fast internal memory and 768 ‘CUDA’ cores. The simulations took between 4–7 hours depending on the complexity of the problem.

## Results and Discussions

**Figure 4:**
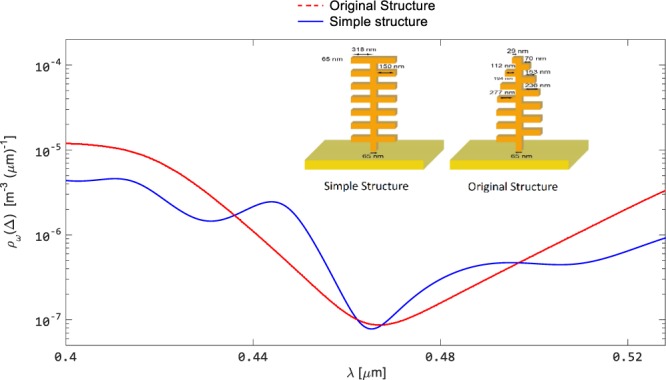
Comparison of the spectral LDOS profiles for ‘simple’ and ‘original’ palm-tree-like structures. The emission profile fr®om the ‘original’ structure (red plot) results in a broader dip at the blue wavelength, whereas in the case of the ‘simple’ structure (blue plot), the dip is sharper and narrower.

**Figure 5:**
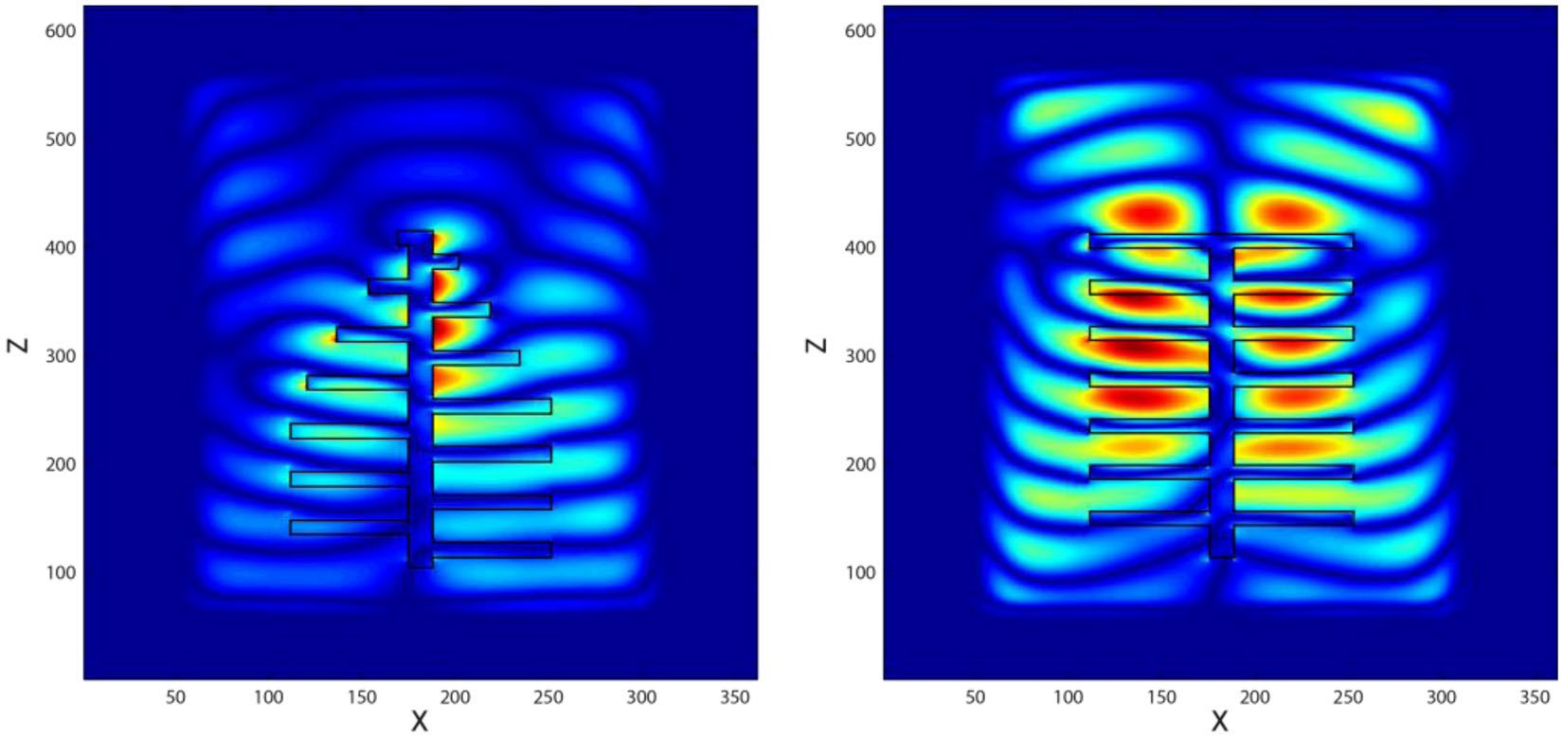
Scattered electric field within the *XZ* plane simulations when the structures are excited by a plane wave at the blue wavelength. Results are for the ‘original’ structure (left) versus the ‘simple’ structure (right) of ‘pine-tree-like-structure’ of *Morpho* butterfly.

Below, we first provide a number of results for the figures of merit. The electric field intensities and the radiative emission profiles are displayed for the ‘simple’ and ‘original’ structures. As reviewed earlier in the Introduction, blue *Morpho* butterfly exhibits a peak in its reflection which is associated with the multilayer periodic interfaces and the scales of the wings^35^. If we assume an opaque structure, we can calculate the absorption profiles, and then determine the emission profile using the Kirchhoff law. It can be observed in Fig. 4 that the emission profile from the ‘original’ structure results in a broader dip at the blue wavelength, whereas in the case of the ‘simple’ structure, the dip is sharper and narrower, which is in agreement with the results given by Siddique *et al*.^45^. The second set of results for the figures of merit are shown in Fig. 5 for the scattered electric field intensity profiles for the ‘simple’ and ‘original’ structures. The scattered electric field intensity is correlated with the measured reflected light in the nanostructures, mimicking the optical active parts of the *Morpho* butterfly wings. It has an intense reflection profile in the case of ‘simple’ structure when compared with the ‘original’ structure, which agrees with those reported^45^. Furthermore, these results reveal that reflection (can be mentioned for scattering as well) patterns for these structures are different from each other and are dependent on the structure of the choice.

After establishing the validity of the results for the benchmark cases and based on our observations, we conducted further simulations for a set of ‘simple’ structures. The radiative emission and heat flux from phononic ‘simple’ structure were evaluated in order to determine their spectral behaviour in mid-infrared. These structures are assumed to be made from a material that supports surface phonon polaritons (SiC). The resonance frequency peak and optical properties of SiC are tuned in mid-infrared^57^. Naturally, such a material exhibits emission peaks within the Reststrahlen band and we can observe an increase in the emission and consequently radiative heat flux profiles, which coincides with the atmospheric transparency window.

**Figure 6:**
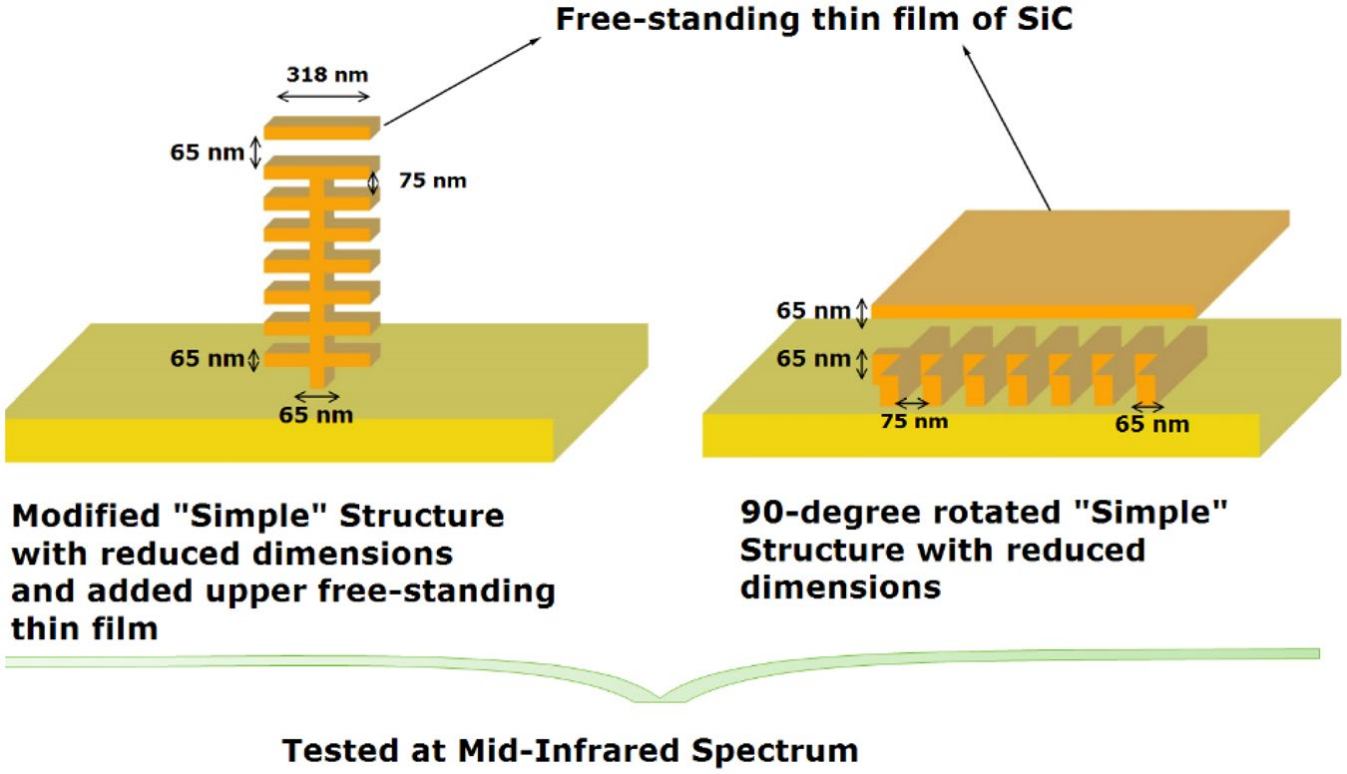
The modified structures with the free-standing film and 90-degree rotated structure. This modified ‘simple’ structure is used for the mid-infrared wavelength spectra calculations. Note that the yellow-colored substrate is not considered in the calculations and is solely displayed for the sake of visualization.

For radiative cooling applications, a surface has to be highly emissive within the atmospheric window and also highly reflective outside of this window. To achieve such ‘design’ conditions, and increase the emissivity within the atmospheric window, we further modified the geometry of the system. For this purpose, we reduced the dimensions of the ‘simple’ structure by decreasing the height of the tree into half and the separation distance between the branches from 150 nm to 75 nm and we introduced an extra free-standing thin film. This thin film is assumed to have the same thickness of 65 nm as the thickness of the tree branches, and it is placed at 65 nm above the first tree-like structure, creating the near-field scenario. This modification increased the magnitude of the near-field emission and heat flux profiles; however, the rate of enhancement was still insignificant. We further modified the system and rotated the ‘simple’ structure by 90 degrees clockwise. Both of these scenarios are demonstrated in Fig. 6. Again, we note that we model only the tree structure, which shows the near field effects due to the proximity of branches to each other and to the thin film above them (as tested at mid-infrared spectrum). The yellow-colored zone is not considered in the calculations and is solely displayed for the sake of visualization.

**Figure 7:**
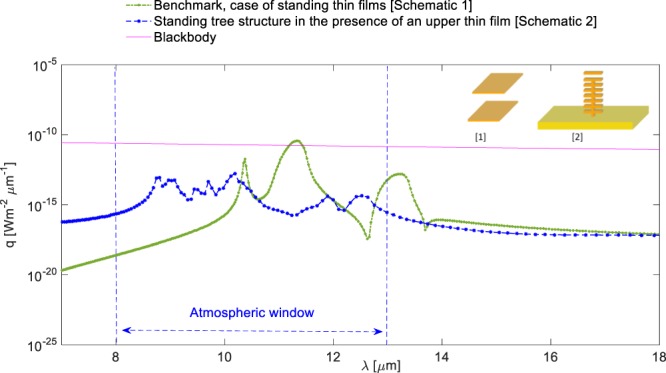
Spectral radiative heat flux profiles at near-field for ‘simple’ structure in the presence of the upper free-standing thin film, compared against the benchmark scenario and the blackbody predictions at temperature T = 1000 K.

**Figure 8:**
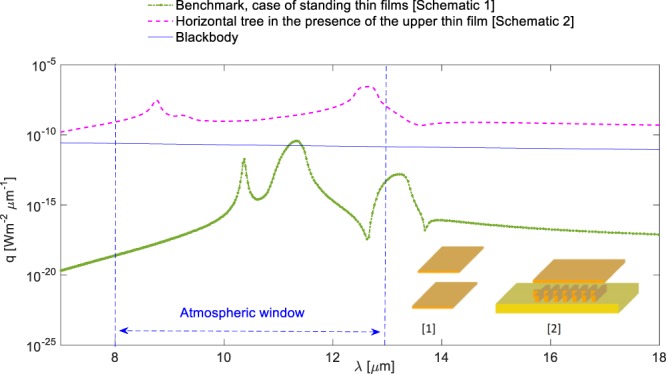
Spectral radiative heat flux profiles at near-field for 90-degree rotated ‘simple’ structure in the presence of the upper free-standing thin film, compared against the benchmark scenario and the blackbody predictions at temperature T = 1000 K. The new geometries display a significant spectral increase (about five orders of magnitude more than the blackbody predictions).

In order to clearly illustrate the achieved enhancement rates and the potential impact of the structural features, we included a well-known benchmark scenario, which is extensively used and analysed in the literature^46,50,53,58^. This is the case of two thin SiC films of 65 nm thickness separated from each other by a vacuum nano-gap of 750 nm and the near-field radiative heat flux is calculated right in the middle of the gap. We obtained the analytical solution for this benchmark scenario. The results given in Figs 6, 7 and 8 clearly depict the enhancement of near-field radiative heat transfer in the modified geometries when compared against the benchmark case and against the blackbody radiation. Figure 7 depicts near-field radiative heat flux from for ‘simple’ structure in the presence of an upper free standing thin film, compared against the benchmark scenario and the blackbody predictions at temperature T = 1000 K. The comparison against the benchmark and blackbody shows that the near-field heat flux within the atmospheric window has to be significantly increased to be considered a good candidate for potential radiative cooling applications. For this purpose, we rotated the ‘simple’ structure by 90 degrees clockwise. As demonstrated in Fig. 8, the magnitude of the near-field radiative heat flux from the rotated structure is increased and surpasses both the blackbody radiation and also the benchmark scenario.

**Figure 9:**
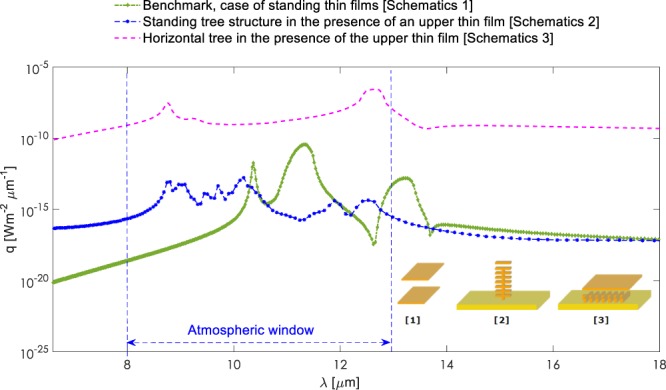
Spectral radiative heat flux profiles at near-field for benchmark scenario of standing thin films, standing tree structure in the presence of an upper thin film and 90-degree rotated ‘simple’ structure in the presence of the upper free-standing thin film. The results show the near-field radiative heat flux is strongly dependent on the geometric features and dimensions. The highest enhancement rates are achieved by the 90-degree rotated structure.

The enhancements observed from the modified structures can be associated with the additional coupling of surface phonon polaritons introduced by the tree branches, the new upper thin film standing on the top of the structures, and also due to the size of the vacuum gap between the standing thin film and the tree structures. These comparisons demonstrate the importance of such complex structures for tuning the near-field radiative transfer emission. In Fig. 9, we compare spectral radiative heat flux profiles at near-field for the benchmark scenario of standing thin films, standing tree structure with thin film, and 90-degree rotated ‘simple’ structure with thin film. The results show that the near-field radiative heat flux is strongly dependent on the geometric features and given dimensions. The highest enhancement rate among the structures considered here is achieved by the 90-degree rotated structure.

In this study, we have only reported the results for SiC, although we have tested various other phononic materials such as hBN, GaAs, InP, α-Fe2O3. Our observations show that radiative heat flux at near-field from ‘simple’ structures can be tuned based on the optical properties of the material of choice and for the desired application. If radiative cooling is emphasized, we can tailor the properties of the system to extend to the second atmospheric transparency window (20–25 μm). This is important, as depending on the specific goals, cooling demands may vary.

## Conclusions

Nature has always been an inspirational source for scientists and researchers of all fields. Studies of the evolution process of many species and organisms have led into unique discoveries in science and technology. In this work, inspired by the magnificent structures of *Morpho didius* butterfly wings, which display rich blue iridescence, we have proposed a biomimicry design that may have the potential to be used in radiative cooling applications at mid-infrared spectrum. This structure which consists of a palm-tree like structure and has multilayer periodic stack of thin films separated by nano-gaps; it is used to create near-field energy exchange in which the free-standing tree structure is rotated 90 degrees clockwise and is brought close to another thin film. The separation distance between the branches and their sizes are changed to see their relative effects. This modified system shows a distinct increase of near-field radiative transfer within the atmospheric window (8–13 μm) when phononic materials such as SiC are used as the material of the choice. These observations suggest that the right combination of these biomimicry designs and materials can be used to tailor radiative heat flux at near-field which may have potential to be used in advanced radiative cooling applications.
