# Publisher Correction: A biomimicry design for nanoscale radiative cooling applications inspired by *Morpho didius* butterfly

**DOI:** 10.1038/s41598-018-37080-x

**Published:** 2019-02-26

**Authors:** Azadeh Didari, M. Pinar Mengüç

**Affiliations:** Center for Energy, Environment and Economy (CEEE), Özyegin University, Istanbul, 34794 Turkey

Correction to: *Scientific Reports* 10.1038/s41598-018-35082-3, published online 15 November 2018

The original PDF version of this Article contained a truncated Figure 4. This error has now been corrected in the PDF version of the Article; the HTML version was correct from the time of publication.

**Figure 4:**
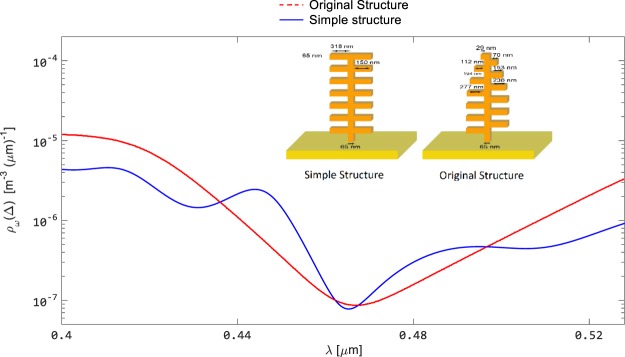
Comparison of the spectral LDOS profiles for ‘simple’ and ‘original’ palm-tree-like structures. The emission profile from the ‘original’ structure (red plot) results in a broader dip at the blue wavelength, whereas in the case of the ‘simple’ structure (blue plot), the dip is sharper and narrower.

